# Validity of the Polar H10 Sensor for Heart Rate Variability Analysis during Resting State and Incremental Exercise in Recreational Men and Women

**DOI:** 10.3390/s22176536

**Published:** 2022-08-30

**Authors:** Marcelle Schaffarczyk, Bruce Rogers, Rüdiger Reer, Thomas Gronwald

**Affiliations:** 1 Department Sports and Exercise Medicine, Institute of Human Movement Science, University of Hamburg, 20148 Hamburg, Germany; 2 Institute of Interdisciplinary Exercise Science and Sports Medicine, MSH Medical School Hamburg, 20457 Hamburg, Germany; 3 Department of Internal Medicine, College of Medicine, University of Central Florida, Orlando, FL 32827, USA

**Keywords:** HRV, RR intervals, DFA a1, chest strap, wearable, endurance exercise

## Abstract

Heart rate variability (HRV) is frequently applied in sport-specific settings. The rising use of freely accessible applications for its recording requires validation processes to ensure accurate data. It is the aim of this study to compare the HRV data obtained by the Polar H10 sensor chest strap device and an electrocardiogram (ECG) with the focus on RR intervals and short-term scaling exponent alpha 1 of Detrended Fluctuation Analysis (DFA a1) as non-linear metric of HRV analysis. A group of 25 participants performed an exhaustive cycling ramp with measurements of HRV with both recording systems. Average time between heartbeats (RR), heart rate (HR) and DFA a1 were recorded before (PRE), during, and after (POST) the exercise test. High correlations were found for the resting conditions (PRE: r = 0.95, r_c_ = 0.95, ICC_3,1_ = 0.95, POST: r = 0.86, r_c_ = 0.84, ICC_3,1_ = 0.85) and for the incremental exercise (r > 0.93, r_c_ > 0.93, ICC_3,1_ > 0.93). While PRE and POST comparisons revealed no differences, significant bias could be found during the exercise test for all variables (*p* < 0.001). For RR and HR, bias and limits of agreement (LoA) in the Bland–Altman analysis were minimal (RR: bias of 0.7 to 0.4 ms with LoA of 4.3 to −2.8 ms during low intensity and 1.3 to −0.5 ms during high intensity, HR: bias of −0.1 to −0.2 ms with LoA of 0.3 to −0.5 ms during low intensity and 0.4 to −0.7 ms during high intensity). DFA a1 showed wider bias and LoAs (bias of 0.9 to 8.6% with LoA of 11.6 to −9.9% during low intensity and 58.1 to −40.9% during high intensity). Linear HRV measurements derived from the Polar H10 chest strap device show strong agreement and small bias compared with ECG recordings and can be recommended for practitioners. However, with respect to DFA a1, values in the uncorrelated range and during higher exercise intensities tend to elicit higher bias and wider LoA.

## 1. Introduction

Heart rate variability (HRV) is believed to reflect autonomic nervous system activity by non-invasively measuring the time and pattern between consecutive R-waves in the electrocardiogram (ECG) [1,2]. It encompasses a wide range of application fields including medical and sport-specific settings [3,4]. Here, studies mainly apply HRV in the context of physical exercise and training monitoring purposes and for the optimization of the training process, mostly by taking resting measurements with focus on standard linear parameters like the root mean square of successive differences (RMSSD) [4,5,6]. The gold standard measure for the quantification of RR intervals and assessment of HRV is the electrocardiogram (ECG) [7]. However, it has become common practice to use mobile systems (e.g., apps, wearables) and chest straps for data recording, storage, analysis and/or export which offers superior practicability with respect to cost, ease to use, portability and interpretation [8,9,10]. However, an evaluation of these applications and sensor devices in different populations is required in order to ensure the validity of the RR interval measurements for the subsequent HRV interpretation [9,11,12].

A frequently used chest strap device is the Polar H10 (Polar Electro Oy, Kempele, Finland) which has already proven validity to assess RR intervals correctly during rest and physical exercise conditions [13]. In this report, there was a substantially better signal quality during high-intensity activities for the Polar H10 in comparison to a 3-lead ECG Holter monitor which led the authors to the conclusion that simple chest strap devices might be recommended as the gold standard for RR interval assessments when strong body movements are present [13]. However, the investigated sample was very homogeneous in its characteristics (healthy, lean, and physically fit volunteers with an average age of 25 years) and the sample size was small (N = 10), so that a generalization of this conclusion should be regarded with caution. A more recent study evaluated the Polar H10 against a photoplethysmography technology (Welltory, New York, NY, USA) and a 12-channel ECG during resting condition in supine and in seated positions [10]. There were no differences observed in the results of the Polar H10 in comparison to the ECG when Kubios HRV software was used to obtain HRV data. Nevertheless, the comparison focused solely on RMSSD and the sample consisted of professional road cyclists, confirming the validity only for these specificities.

In addition to the requirement to perform population-based validations, to date no validation has been conducted for non-linear measures of HRV. It is possible that agreement with ECG measurement is also dependent on the particular index investigated, which is why there is also the need for metric-specific validations [9]. The non-linear short-term scaling exponent alpha 1 of Detrended Fluctuation Analysis (DFA a1) has shown its suitability to describe the complex cardiac autonomic regulation during various exercise intensities, modalities, and environmental conditions while possessing a wide dynamic range throughout all intensity zones in contrast to standard linear parameters [14,15]. In this context, recent studies show the potential of this parameter to demarcate physiological threshold boundaries by means of fixed DFA a1 values at the aerobic (~0.75) and anaerobic threshold (~0.5) for usage in performance monitoring and exercise intensity distribution in endurance sports [16,17]. Additionally, exercise associated DFA a1 dynamics may also give insights about physiological status in terms of monitoring of fatigue during low-intensity exercise [18]. Therefore, the importance of a validation study with focus on the recording method and DFA a1 becomes evident, since researchers and recreational athletes frequently use this device for data collection and therefore rely on appropriate measurements.

The present study compared selected HRV data (with inclusion of DFA a1) obtained by the Polar H10 chest strap device using the Elite HRV application for data recording, storage and export vs. a 12-channel ECG and analyzed by Kubios HRV Software version 3.5.0 (Biosignal Analysis and Medical Imaging Group, Department of Physics, University of Kuopio, Kuopio, Finland [19]) during resting and exercise conditions in a group of female and male recreational athletes.

## 2. Materials and Methods

### 2.1. Participants

**Figure 1:**
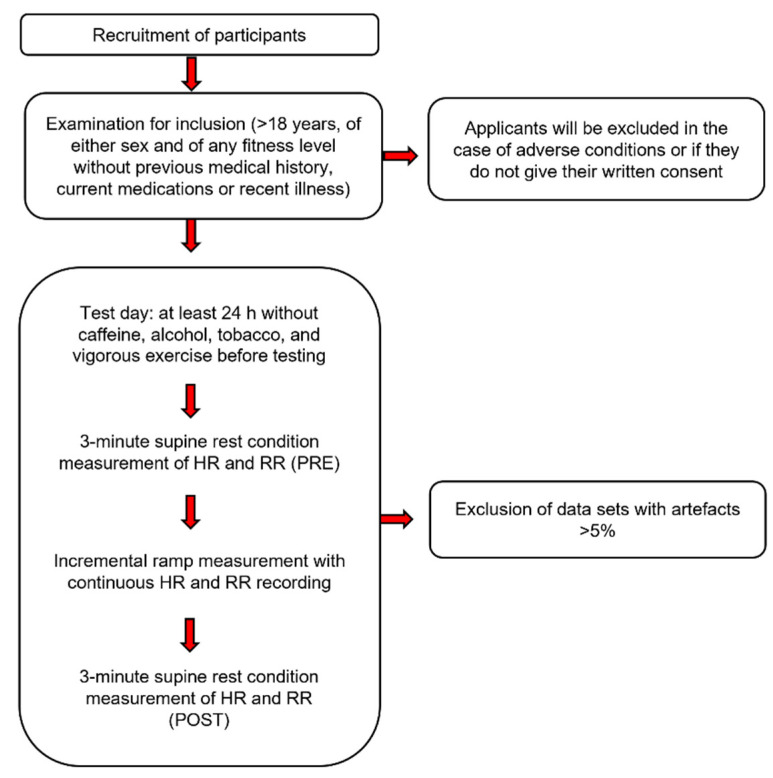
Flow chart of the methodical procedure.

Twenty-five participants (men: n = 14, age: 40 ± 14 years, height: 178.1 ± 9.0 cm, body weight: 82.2 ± 14.8 kg; women: n = 11, age: 34 ± 10 years, height: 169.1 ± 4.3 cm, body weight: 67.8 ± 9.5 kg) volunteered to take part in this study after being recruited via word of mouth as via the internet. Entry criteria included adults (>18 years) of either sex and of any fitness level without previous medical history, current medications or recent illness. Participants were asked to abstain from caffeine, alcohol, tobacco, and vigorous exercise 24 h before testing and provided written informed consent. Ethical approval for the study was obtained by the University of Hamburg, Department of Psychology and Movement Science, Germany (reference no.: 2021_400) and is in line with the principles of the Declaration of Helsinki. The complete methodical procedure can be seen in Figure 1.

### 2.2. Exercise Protocol and Data Acquisition

**Figure 2:**
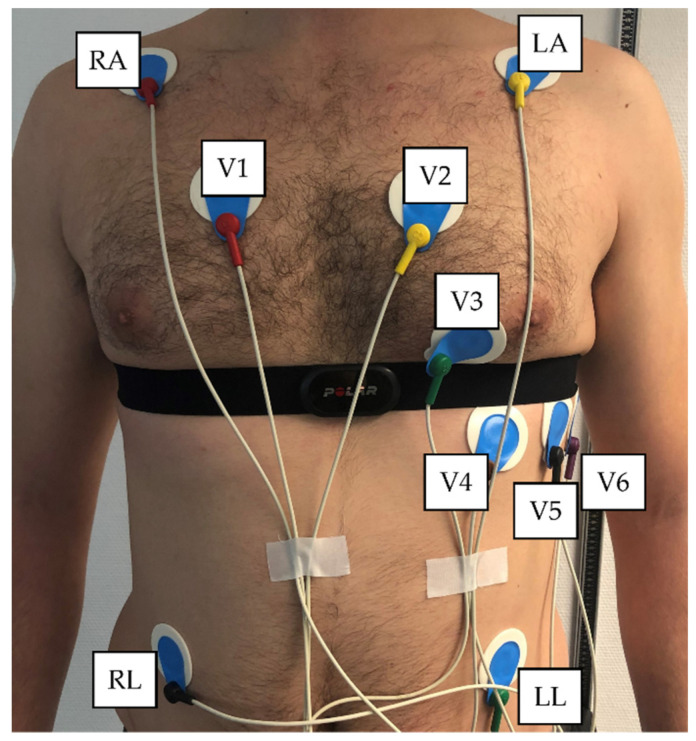
Placement of 12-channel ECG electrodes and Polar H10 chest strap device. V1 = 4th intercostal space at the right border of the sternum, V2 = 4th intercostal space at the left border of the sternum, V3 = midway between locations V2 and V4, V4 = at the mid-clavicular line in the 5th intercostal space, V5 = at the anterior axillary line in the same horizontal level as V4, V6 = at the mid-axillary line on the same horizontal level as V4 and V5. Limb leads of the right arm (RA) and left arm (LA) outwardly on the shoulders and right leg (RL) and left leg (LL) leads at the lower edge of the ribcage.

An incremental ramp was performed on a mechanically braked cycle (Ergoselect 4 SN, Ergoline GmbG, Bitz, Germany) with cadence kept between 60–80 rpm. The protocol consisted of a three-minute initial workload of 50 Watts followed by a 1 watt increase every 3.6 s (equivalent to 50 Watts/3 min) until the volunteer’s voluntary exhaustion. Recordings of heart rate (HR) and RR intervals were taken continuously during the exercise test, as well as prior (PRE; after a period of habituation and session preparation) and post (POST) exercise by means of 3-min supine rest condition measurement intervals with two devices at the same time: (1) 12-channel ECG CardioPart 12 Blue (AMEDTEC Medizintechnik Aue GmbH, Germany; sampling rate: 500 Hz; desktop software: AMEDTEC ECGpro version 5.10.002), and (2) Polar H10 sensor chest strap device (Polar Electro Oy, Kempele, Finland; sampling rate: 1000 Hz; app software: Elite HRV App, Version 5.5.1). Placement of the ECG electrodes and chest strap device is pictured in Figure 2.

Breath-by-breath pulmonary gas exchanges were recorded throughout the ramp using a metabolic analyzer (Quark CPET, module A-67-100-02, COSMED Deutschland GmbH, Fridolfing, Germany; desktop software: Omnia version 1.6.5). Prior to testing, the gas analyzers were calibrated according to the manufacturer’s instructions. The protocol was terminated when the participant fell below a cadence of 60 rpm or due to self-determination. The following criteria served for the assumption of exhaustion: (A) heart rate >90% of the maximum predicted heart rate (prediction model according to [20]: 208 − (0.7 × age) and (B) respiratory quotient > 1.1. Maximum oxygen uptake (VO_2max_) and maximum HR (HR_max_) were defined as the average VO_2_ and HR over the last 30 s of the test.

### 2.3. Data Processing

After extraction of the 12-channel ECG data (converted from exported.xml files) and RR data (exported from the Elite HRV app) as text files, import into Kubios HRV Premium Software version 3.5.0 (Biosignal Analysis and Medical Imaging Group, Department of Physics, University of Kuopio, Kuopio, Finland, [19]) was conducted. For the 12-channel ECG, lead 2 was used as the comparable lead to the chest strap device [21]. Preprocessing settings were set to the default values including the RR detrending method which was kept at “smoothness priors” (Lambda = 500). The RR series was then corrected by the Kubios HRV Premium “automatic method” [22]. For DFA a1 calculation window width was set to 4 ≤ n ≤ 16 beats [23]. During rest conditions a 2-min time window (00:30–02:30 min:s) was chosen for the analysis. During the incremental exercise time-varying analysis was adjusted to a 2-min window width and 20-s grid interval for the moving window, so that the exported.csv files from Kubios HRV Premium Software contained the HRV metrics of interest (RR, HR, DFA a1) recalculated every 20 s. Data sets with artefacts >5% were excluded from analysis based on [24].

### 2.4. Statistics

Results are presented as mean ± standard deviation (SD). Normal distribution of data was checked by Shapiro–Wilk testing and visual inspection of data histograms. Agreement of the variables obtained by means of the two devices during the three conditions (PRE, incremental exercise, POST) was evaluated using linear regression, Pearson’s r correlation coefficient (r), Lin’s Concordance Correlation Coefficient (r_c_), Intraclass Correlation Coefficient (ICC_3,1_), coefficient of determination (R^2^), standard error of estimate (SEE) and Bland–Altman plots with limits of agreement (LoA) [25]. The size of Pearson’s r correlation coefficient was evaluated as follows; 0.3 ≤ r < 0.5 low; 0.6 ≤ r < 0.8 moderate and r ≥ 0.8 high [26]. Computation of r_c_ was conducted using a S1Macro for SPSS (https://doi.org/10.1371/journal.pone.0239931.s002 (accessed on 5 July 2022). It represents a modification of Pearson’s r correlation coefficient, in that it assesses not merely the distance of data points to the line of best fit, but also how far this line deviates from the line of perfect agreement, as represented by the 45-degree line through the origin. Sizes of ≥0.8 are rated at almost perfect agreement [27]. Bland–Altman mean differences for data comparisons were expressed as either absolute or percentage bias (difference/mean × 100). Analyze-it software (Version 5.66) was used for its automatic computation. In the case of normal distribution, paired t-test was used for the comparison of data, whereas Wilcoxon Signed Ranks Test was applied in case of violation of the precondition. In the case of non-normally distributed data, medians and estimates of the median differences along with the 95% confidence intervals (Hodges-Lehmann estimator) were additionally calculated using a SPSS Syntax code (Hodges-Lehmann Confidence Interval for Median difference|Raynald’s SPSS Tools; [28]). Effect sizes were calculated with Cohen’s d (d) and its respective thresholds (small effect = 0.20, medium effect = 0.50, large effect = 0.80; [29]. For all tests, the statistical significance was accepted as *p* ≤ 0.05. Analysis was performed using IBM^®^ SPSS^®^ Statistics 25 and Microsoft Excel 365 (Version 2204).

## 3. Results

The participants reached maximal power (P_max_) values of 258 ± 49 watts, a VO_2max_ of 40.6 ± 7.6 mL/kg/min and a HR_max_ of 178 ± 14 bpm (men: 279 ± 51 watts, 42.1 ± 8.8 mL/kg/min, 180 ± 17 bpm; women: 230 ± 31 watts, 38.7 ± 5.6 mL/kg/min, 177 ± 10 bpm). Five participants were excluded from exercise analysis and two participants from resting POST measurement due to artefacts >5%.

### 3.1. PRE and POST Analysis

**Figure 3:**
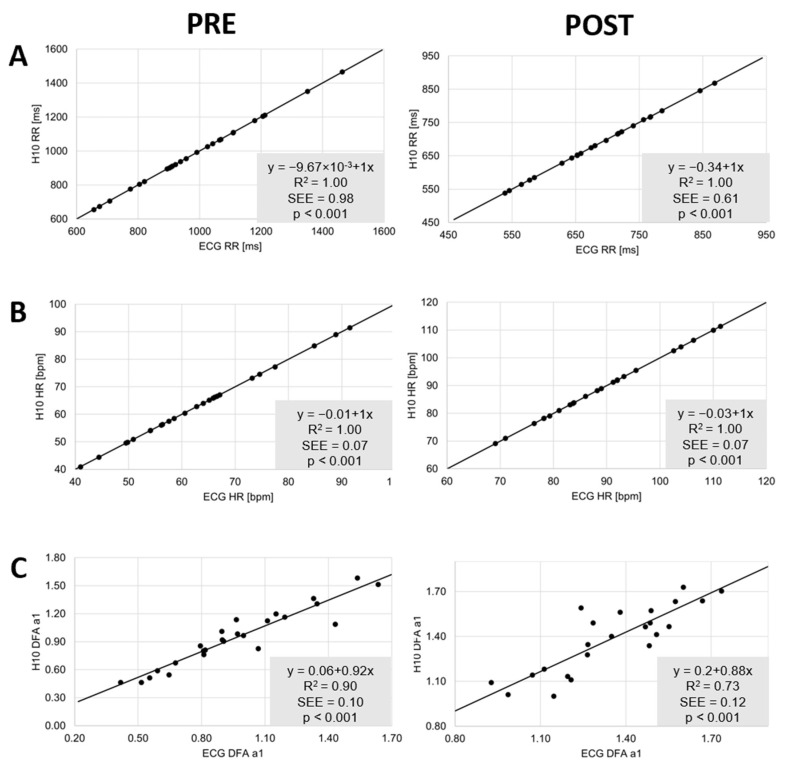
Regression plots for the comparison of the ECG (ECG) and the Polar H10 sensor chest strap device (H10) during PRE and POST the incremental exercise test for RR (**A**), HR (**B**) and DFA a1 (**C**). Slope, coefficient of determination (R^2^), standard error of estimate (SEE), and *p*-value shown in the bottom right plot.

**Figure 4:**
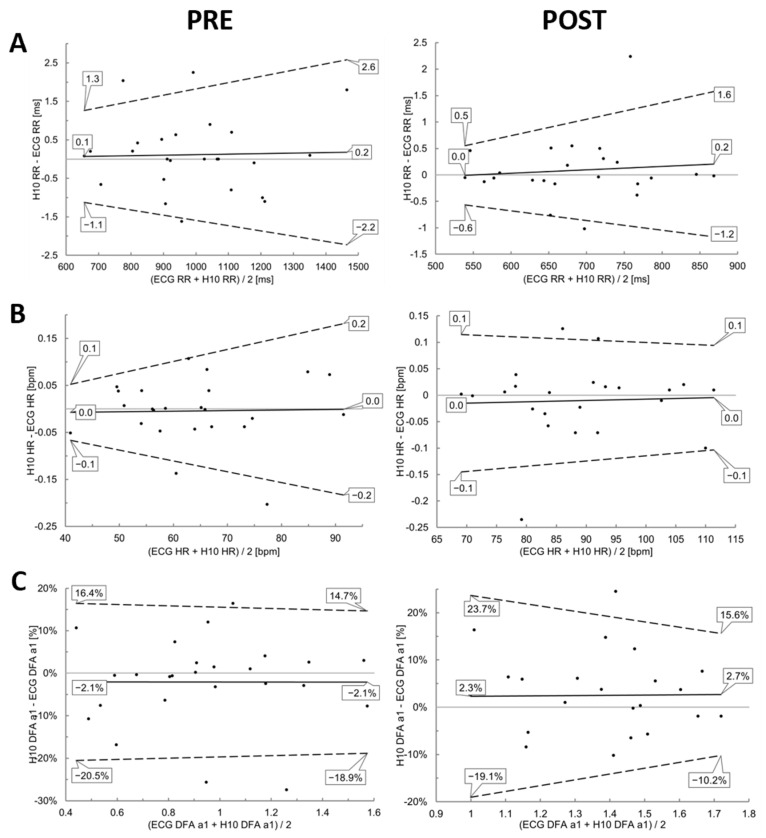
Bland–Altman analysis for the comparison of the ECG (ECG) and the Polar H10 sensor chest strap device (H10) during PRE and POST the incremental exercise test for RR (**A**), HR (**B**) and DFA a1 (**C**). Center solid line in each plot represents the mean bias (difference) between each paired value as absolute (RR, HR) or relative values (DFA a1). The top and bottom dashed lines are 1.96 standard deviations from the mean difference.

The comparisons during resting state conditions PRE and POST showed nearly perfect correlations for the linear parameters RR and HR (r = 1.00, r_c_ = 1.00, ICC_3,1_ = 1.00). Although correlations were also high for DFA a1 (r > 0.86, r_c_ > 0.84, ICC_3,1_ > 0.85), they were comparatively weaker (see Table 1 and Figure 3). There were no significant differences for both conditions and the three variables of interest (*p* > 0.05) (Table 1). Bland–Altman analysis revealed a mean difference of 0.2 to 0.1 ms for RR with upper LoA of 2.6 to 1.3 ms and lower LoA of −2.2 to −1.1 ms in PRE, and a bias of 0.2 to 0.0 ms with upper and lower LoA of 1.6 to 0.5 ms and −1.2 to −0.6 ms in POST, respectively. With respect to DFA a1 there was bias of −2.1% (upper LoA of 14.7 to 16.4% and lower LoA of −18.9 to −20.5%) and 2.5% for POST (upper LoA of 15.6 to 23.7% and lower LoA of −10.2 to −19.1%). Detailed plot analysis for all variables can be found in Figure 4.

### 3.2. Incremental Exercise Analysis

**Figure 5:**
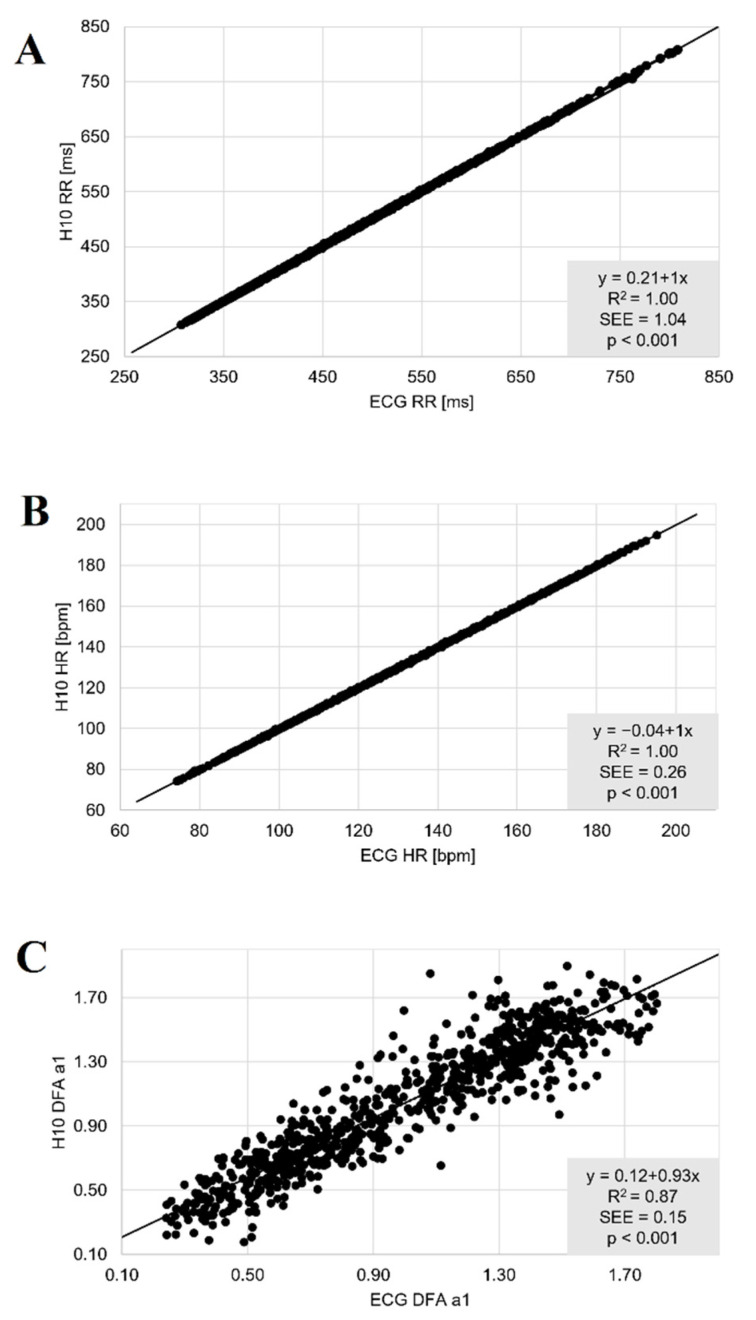
Regression Plots for the comparison of the ECG (ECG) and the Polar H10 sensor chest strap device (H10) during the incremental exercise test for RR (**A**), HR (**B**) and DFA a1 (**C**). Slope, coefficient of determination (R^2^), standard error of estimate (SEE), and *p*-value are shown in the bottom right of each plot.

**Figure 6:**
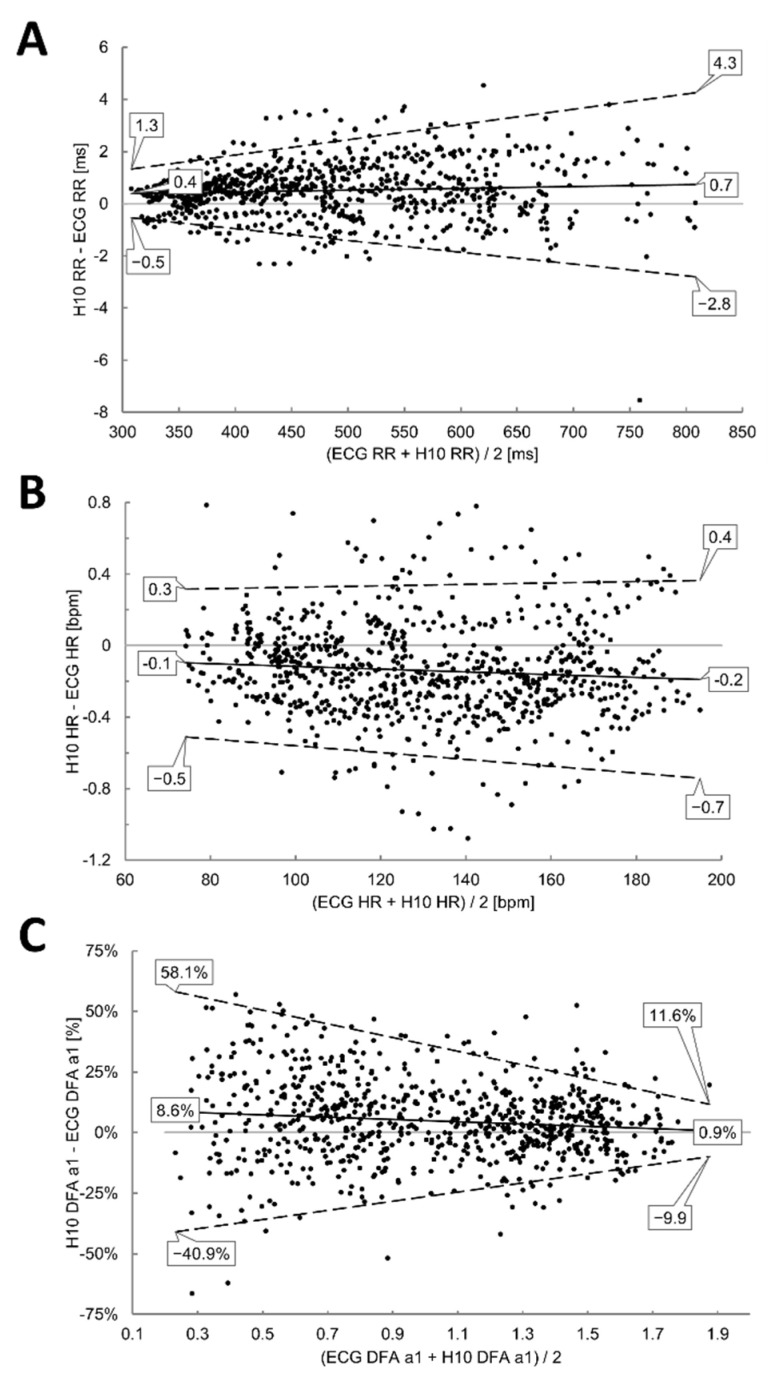
Bland–Altman analysis for the comparison of the ECG (ECG) and the Polar H10 sensor chest strap device (H10) during the incremental exercise test for RR (**A**), HR (**B**) and DFA a1 (**C**). Center solid line in each plot represents the mean bias (difference) between each paired value as absolute (RR, HR) or relative values (DFA a1). The top and bottom dashed lines are 1.96 standard deviations from the mean difference.

High correlations could be found during the incremental exercise test for the linear parameters (r = 1.00. r_c_ = 1.00, ICC_3,1_ = 1.00), and DFA a1 (r > 0.93. r_c_ > 0.93, ICC_3,1_ > 0.93), although with a statistically significant difference (*p* < 0.001) (see Figure 5 and Table 2). Bland–Altman plot for RR showed a mean difference of 0.7 to 0.4 ms with upper and lower LoA of 4.3 to −2.8 ms during low intensity and 1.3 to −0.5 ms during high intensity exercise. DFA a1 showed wider bias (0.9 to 8.6%) and LoAs of 11.6 to −9.9% during low intensity and 58.1 to −40.9% during high intensity. Further results are depicted in Figure 6.

## 4. Discussions

**Figure A1:**
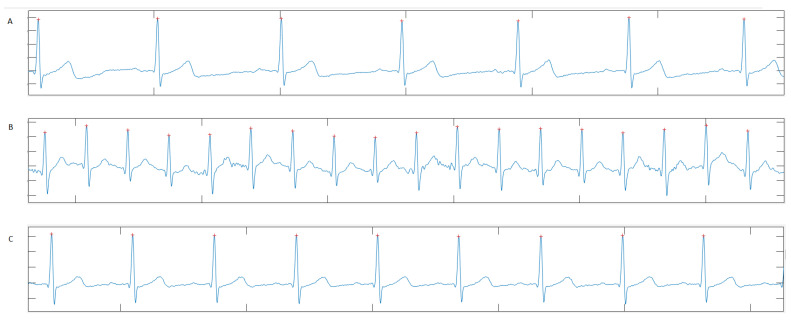
**Figure A1**. Representative participant ECG waveforms and background noise during 6 s windows using Kubios HRV software Premium. (**A**) Pre incremental ramp, HR of 65 bpm. (**B**) During incremental ramp just prior to exhaustion, HR of 180 bpm. (**C**) Post incremental ramp, HR of 92 bpm.

The objective of the present study was to evaluate the extent of agreement of HRV data obtained via the Polar H10 sensor chest strap device with that recorded by means of a reference laboratory grade ECG. Comparisons were done for RR intervals, HR and DFA a1 both at resting conditions (PRE and POST) and during incremental cycling exercise (see Figure A1 for exemplary ECG waveforms). Data for linear HRV showed excellent, near perfect agreement during resting conditions with the divergences being minimal and not clinically relevant. Considering DFA a1 comparison, while mean bias was −2.1% for PRE (upper LoA of 14.7 to 16.4% and lower LoA of −18.9 to −20.5%) and 2.5% for POST (upper LoA of 15.6 to 23.7% and lower LoA of −10.2 to −19.1%) in the present study, it was 1.0% (LoA: 20.5 to −18.5 %)) and −0.7% (LoA: 14.2 to −15.7%) in a comparable chest strap device validation with the Movesense Medical Sensor [30]. Although comparisons where statistically different during the incremental exercise test, absolute mean bias in RR was very low (under 1 ms), LoA were close (upper and lower LoA of 4.3 to −2.8 ms during low intensity and 1.3 to −0.5 ms during high intensity exercise) and correlation coefficients were high (from 0.93 to 1.0). Magnitude of DFA a1 bias was small with low intensity showing a percentage difference of 0.9% (LoA: 11.6 to −9.9%) and an increased difference of 8.6% (LoA: 58.1 to −40.9%) during high intensity exercise, comparable to the study by [30] with the Movesense Medical sensor device.

Since DFA a1 has been employed as a marker of both physiological thresholds and autonomic fatigue during exercise [15,18], the clinical consequence of DFA a1 divergence between devices should be examined. Index values tended to be approximately 6% and 7.5% higher at the fixed values of 0.75 and 0.5 for Polar H10 in comparison to the ECG (Figure 5C), with expected consequences for the proposed HRV threshold boundaries [15]. Both threshold boundaries were crossed at higher HRs when using the Polar H10 which although are relatively minor, could have consequences for exercise and training prescription and detection of fatigue. However, the amount of deviation was variable, with some participants showing very close agreement between devices. The degree of bias may simply relate to the discordance between actual ECG waveform signal strength and morphology [21]. As previously noted with the Movesense Medical sensor as a single channel ECG module [30], some individuals will exhibit variation in DFA a1 through an exercise ramp simply based on the difference between a chest strap sensor vs. a lead 2 ECG waveform. Hence, it is certainly plausible that some participants had either different or sub optimal signal strength using a chest strap device as opposed to ECG lead 2. In fact, Polar’s own documentation recommends that both strap placement and even module inversion be considered if results appear unreliable [31]. Therefore, it is recommended to visually evaluate an initial baseline of the Polar H10 ECG waveform before usage for exercise intensity investigation. This can help one optimize QRS wave morphology by chest strap rotation and/or module inversion [32] for best signal to noise ratio. Several smartphone apps are available providing ECG waveform display and recording including Fatmaxxer (https://github.com/IanPeake/FatMaxxer) and the Polar Equine app (https://play.google.com/store/apps/details?id=fi.polar.equine&gl=US).

Other factors may also affect RR time series accuracy, thereby impacting DFA a1 with its reliance on actual patterns of RR intervals over time [33]. Unfortunately, hardware and software technicalities are often just known by the manufacturers themselves, despite the widespread knowledge of the effects of, e.g., sampling frequency, RR resolution or the preprocessing algorithms used for filtering background noise or muscular contractile activity on RR intervals [1,12,34,35]. Although both the Polar H10 and ECG based RR measurements are computed with signal processing algorithms resembling the Pan-Tompkins method, the exact rules and procedures are likely different, leading to some degree of end result deviation. In addition, factors involving the individual characteristics of the male and female participants (ventricular size or mass, subcutaneous fat, skin characteristics), electrode positioning, choice of lead for analysis or cardiac disease pathology might affect the QRS complex and thus the timing of the RR detection [1,11,21,36,37,38,39]. As discussed above, the recording systems compared here not only differ in device type, but as mentioned above also in lead placement, therefore it is impossible to attribute a single variable for the data discrepancy.

**Figure 7:**
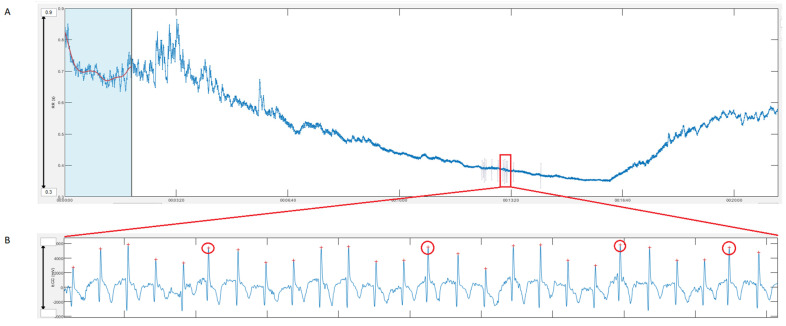
(**A**) Modified Kubios HRV Premium software output of the raw data of the Polar H10 in one female participant during incremental cycling ramp; the measurement window from minute 12 to 14 indicated artifacts over 6%. (**B**) Evaluation of the lead 2 ECG waveform recordings during the same measurement window, there could be found some runs of atrial premature complexes (APC, red circles) pointing to the artifacts not really being artifacts and the pitfall of a mere RR recording.

Although not directly related to the issue of HRV agreement, mention should be made of the disadvantages of just recording RR intervals without continuous ECG tracings. Since the Polar H10 only stores RR intervals and not ECG waveform recordings, it is not possible to identify or potentially correct signals with excessive artefacts leading to a data loss. In this context, it is also impossible to differentiate whether abundant artifacts originate from noise or due to cardiac arrhythmia [30]. Figure 7 illustrates this with an example of a female participant from the present study whose data during the incremental cycling ramp was excluded from validation analysis since artifact exceeded >5%. On closer examination of the parallel recorded ECG, these “artifacts” were classified as frequent atrial premature complexes (APCs) which can be a risk factor for atrial fibrillation [40]. In terms of DFA a1 bias during the incremental tests, artifact correction can lead to variable results [24]. 

**Figure 8:**
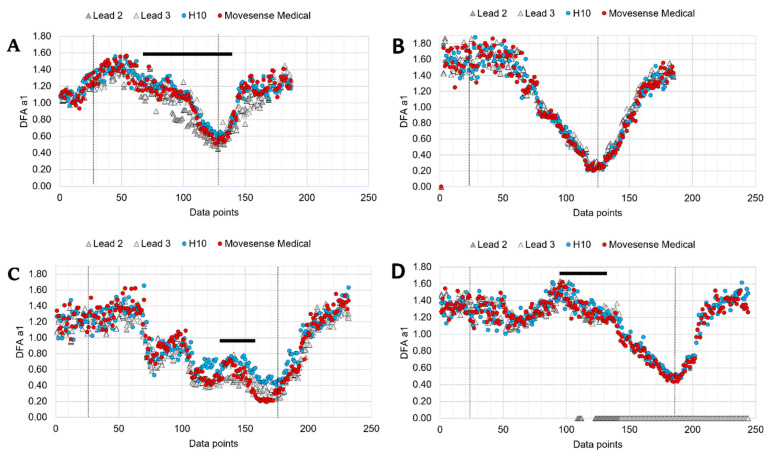
Course of DFA a1 during incremental cycling ramp until voluntary exhaustion, including 3 min of warm-up at 50 W and 5 min cool-down of unloaded pedaling (vertical lines mark the start of the incremental test and voluntary exhaustion) wearing a 12-channel ECG (lead 2 and 3 depicted), a Polar H10 sensor chest strap device, and a Movesense Medical sensor single channel ECG chest strap device. (**A**) Good agreement between both chest strap devices and ECG lead 3 in a male participant with ECG lead 2 showing up to 50% divergence from the Polar H10 sensor (artifacts 2% during the marked range; black line). (**B**) Female participant with excellent agreement in all four signal sources. (**C**) Female participant showing high artifacts (>5%) in the marked range (black line) and deviations between all 4 devices. (**D**) Obese participant with artifacts >5% in the marked range (black line) for ECG Lead 2 and 3 with a subsequent complete loss of the signals. However, minimal artefacts in Polar H10 and Movesense Medical sensor (<1%).

Even though this study attempted to exclude recordings exceeding 5% artifact, it cannot be guaranteed that sporadic DFA a1 variation did not occur. This can be observed in Figure 8 where DFA a1 courses of four study participants are depicted. The effects of high degrees of artifact correction are demonstrated, showing either data loss or HRV bias. The potential effect of lead placement (chest strap electrodes vs. ECG lead 2) is also notable in terms of DFA a1 determination.

It can be considered as a possible limitation that this study did not group the samples by their individual personal characteristics. Future studies could elucidate if body composition, gender, age or ventricular characteristics influence study outcomes. For recreational athletes with the aim of using the Polar H10 chest strap device with the Elite HRV App, it should be emphasized that raw data output assuming no data loss or corrections was analyzed via Kubios HRV Premium software containing the feature of automatic artifact correction. The basic, cost-free “standard” version of Kubios HRV with an alternate threshold-based artifact correction method could lead to slightly different results than presented [24].

## 5. Conclusions

Linear HRV measurements derived from the Polar H10 sensor chest strap device recorded with the Elite HRV app correspond closely with measurements taken with a reference ECG in terms of RR intervals and HR. However, with respect to DFA a1, values in the uncorrelated range and during higher exercise intensities tend to elicit higher bias and wider LoA. This may partly be related to the expected differences in non-linear HRV associated with ECG lead placement. Nevertheless, since this mobile-based HRV recording setup displays superior practicability with generally comparable results, its commercial use for the monitoring of HRV data during resting and endurance exercise conditions could be justified.

## Figures and Tables

**Table 1:** Mean, standard deviation (SD), Minimum and Maximum for RR, HR and DFA a1 obtained from the ECG (ECG) and the Polar H10 sensor chest strap device (H10) during resting conditions. Statistics are represented by means of *p*-value, Cohen’s d (d), Pearson’s r (r), Lin’s Concordance Correlation Coefficient (r_c_) and Intraclass Correlation Coefficient (ICC_3,1_).

	PRE						POST					
	RR [ms]		HR [bpm]		DFA a1		RR [ms]		HR [bpm]		DFA a1	
	H10	ECG	H10	ECG	H10	ECG	H10	ECG	H10	ECG	H10	ECG
mean	987.6	987.5	63.2	63.2	0.94	0.96	686.4	686.3	88.9	88.9	1.38	1.35
SD	201.1	201.1	13.1	13.1	0.31	0.32	90.5	90.4	11.9	11.9	0.23	0.22
*p*-value	0.56		0.74		0.35		0.50		0.50		0.18	
d	0.12		0.07		0.19		0.14		0.14		0.29	
r	1.00		1.00		0.95		1.00		1.00		0.86	
r_c_	1.00		1.00		0.95		1.00		1.00		0.84	
ICC_3,1_	1.00		1.00		0.95		1.00		1.00		0.85	

**Table 2:** Mean, standard deviation (SD) and medians for RR, HR and DFA a1 obtained from the ECG (ECG) and the Polar H10 sensor chest strap device (H10) during the incremental exercise test. Paired data statistics are represented by means of Hodges-Lehmann estimator, *p*-value, Cohen’s d (d), Pearson’s r (r), Lin’s Concordance Correlation Coefficient (r_c_) and Intraclass Correlation Coefficient (ICC_3,1_).

	RR [ms]		HR [bpm]		DFA a1	
	H10	ECG	H10	ECG	H10	ECG
mean	485.5	485.0	130.1	130.2	1.05	1.01
SD	112.4	112.3	28.7	28.7	0.40	0.40
median	470.0	470.3	127.7	127.6	1.07	1.01
Hodges-Lehmann estimator	−0.48 (−10.75 to 9.74)		−0.14 (−2.75 to 3.04)		−0.04 (−0.08 to 0.00)	
*p*-value	<0.001		<0.001		<0.001	
d	0.48		0.51		0.28	
r	1.00		1.00		0.93	
r_c_	1.00		1.00		0.93	
ICC_3,1_	1.00		1.00		0.93	

## Data Availability

The datasets analyzed during the current study are available from the corresponding author on reasonable request.

## Abbreviations

HRV = heart rate variability, ECG = electrocardiogram, DFA a1 = short-term scaling exponent alpha1 of Detrended Fluctuation Analysis, RR = average time between heartbeats, HR = heart rate, RMSSD = root mean square of successive differences, VO_2max_ = maximum oxygen uptake, r = Pearson’s r correlation coefficient, r_c_ = Lin’s Concordance Correlation Coefficient, ICC_3,10_ = Intraclass Correlation Coefficient, R^2^ = coefficient of determination, SEE = standard error of estimate; LoA = limits of agreement, SD = standard deviation (SD), H10 =Polar H10 sensor chest strap (H10), d = Cohen’s d, APC = atrial premature complex.
